# IMPROVEMENT IN OXIDATIVE STRESS AFTER DUODENOJEJUNOSTOMY IN AN EXPERIMENTAL MODEL OF TYPE 2 DIABETES MELLITUS

**DOI:** 10.1590/0102-6720201600S10002

**Published:** 2016

**Authors:** Cacio Ricardo WIETZYCOSKI, João Caetano Dallegrave MARCHESINI, Sultan AL-THEMYAT, Fabiola Shons MEYER, Manoel Roberto Maciel TRINDADE

**Affiliations:** Master in Surgical Sciences Program, Federal University of Rio Grande do Sul - UFRGS, Porto Alegre, RS, Brazil.

**Keywords:** Diabetes Mellitus, type 2, Oxidative stress, Streptozotocin

## Abstract

**Background:**

Type 2 Diabetes Mellitus is a multifactorial syndrome with severe complications. Oxidative stress is accepted as a causal factor of chronic complications

**Aim:**

To demonstrate alterations in oxidative stress after metabolic surgery.

**Methods:**

Twenty-four 2-day-old Wistar rats were used. In 16, Type 2 Diabetes Mellitus was induced by 100 mg/kg streptozotocin injection. The development of diabetes was confirmed after 10 weeks using an oral glucose tolerance test. Eight diabetic rats composed the diabetic surgical group; the remaining eight composed the diabetic group. Eight animals in which diabetes was not induced formed the clinical control group. The Marchesini technique was used in the diabetic surgical group. After 90 days, the rats were sacrificed, and the oxidative stress markers were measured.

**Results:**

Thiobarbituric acid reactive substances, superoxide dismutase and catalase were significantly reduced in the diabetic surgical group compared to the diabetic group.

**Conclusion:**

The duodenojejunostomy was effective in controlling the exacerbated oxidative stress present in diabetic rats.

## RESUMO

**Racional:**

Diabete melito tipo 2 é síndrome multifatorial com complicações graves. O estresse oxidativo é aceito como um fator causal de complicações crônicas.

**Objetivo:**

Demonstrar alterações no estresse oxidativo após a cirurgia metabólica.

**Métodos:**

Foram utilizados 24 ratos Wistar de dois dias de idade. Em 16, diabete melito tipo 2 foi induzida por 100 mg/kg de injeção de estreptozotocina. O desenvolvimento do diabete foi confirmado após 10 semanas, utilizando teste oral de tolerância à glucose. Oito ratos diabéticos compuseram o grupo cirúrgico diabético; os oito restantes constituíram o grupo diabético. Oito animais em que não foi induzido o diabete formaram o grupo controle clínico. A técnica de Marchesini foi utilizada no grupo cirúrgico diabético. Após 90 dias, os ratos foram sacrificados, e os marcadores de estresse oxidativo foram medidos.

**Resultados:**

Ácido tiobarbitúrico, superóxido dismutase e catalase foram significativamente reduzidos no grupo cirúrgico diabético quando comparado ao grupo diabético.

**Conclusão:**

O duodenojejunostomia foi eficaz no controle do estresse oxidativo exacerbado presente em ratos diabéticos.

## INTRODUCTION

Diabetes Mellitus (DM) is a disease with a high incidence and prevalence in many parts of the world, particularly in the United States and Europe. In Brazil and in other developing countries, the number of DM patients is increasing considerably^28^. The increase incidence in Brazil is an important public health problem because the disease affects various body systems, with frequent complications arising from tissue and vascular alterations as manifested in peripheral vascular disease, ischemic heart disease and cerebrovascular diseases. The number of people with DM is estimated to increase from 150 million in 2000 to 220 million in 2010 and 300 million in 2025^26^. In addition, the estimation of world DM prevalence for all age groups was 2.8% in 2000 and is projected to be 4.4% in 2030^26^. If obesity prevalence stops increasing and remains stable until 2030, which is unlikely, the number of people with DM will be more than the double the number today due to aging and population urbanization. It is likely that these are underestimations of the actual values; data from the International Diabetes Federation indicate that approximately 246 million people in the world have DM, making it one of the most common non-transmissible diseases^24^. In Brazil, according to data from the DM and Systemic Arterial Hypertension Information System of the Ministry of Health, six million diabetics are estimated to exist, half of which are followed-up in health basic units^4^. There is a growing interest in experimental research aimed at investigating the main physiopathological pathways in DM, particularly those contributing to the disease's chronic complications^2^ ^,^ ^11^ ^,^ ^17^ ^,^ ^21^.

Oxidative stress is accepted as a causal factor of chronic complications and may be measured based on the levels of thiobarbituric acid reactive substances (TBARS) and the activity of catalase. Recently, metabolic surgery has garnered increasing interest as an effective option for treating type 2 DM^13^ ^,^ ^14^ ^,^ ^22^. Its remission after metabolic surgery can be explained by theories such as the exclusion of food passage through the duodenum and the proximal portion of the jejunum, known as the foregut theory. On the other hand, the deviation of the proximal small intestine exposes the distal ileum prematurely to nutrients, increasing the secretion of glucagon-like peptide-1 and peptide YY^22^. This early stimulus of food in the ileum, leading to the production of local intestinal hormones, is called the hindgut theory. Surgical techniques developed for diabetes treatment, based on these two theories, either remove the duodenum from intestinal transit, allow food to arrive at the distal ileum earlier, or use both mechanisms. These techniques should be easily performed and safe for the patient^7^ ^,^ ^25^.

This study provides an evaluation of the repercussion of a metabolic surgery (end-to-side duodenojejunostomy described by Marchesini^7^) in Streptozotocin-induced diabetic rats during the neonatal period and its effects on duodenojejunostomy oxidative stress status.

## METHODS

### Experimental animals

The study was performed at the Unit of Animal Experimentation at the Hospital de Clínicas de Porto Alegre following the guidelines of the OMS Ethical Code for Animal Experimentation and approved by the Research Ethics Committee of this institution. Twenty-four approximately 2-days-old male Wistar rats were used, bred in the experimentation unit. After weaning, the animals were kept in a light/dark cycle (12/12 h) in a controlled temperature environment (22±2° C) with a rat-specific food (Purina Rat Chow^(r))^ and water ad libitum.

### Experimental Diabetes Mellitus type 2 induction

In 16 animals, 100 mg/kg of streptozotocin in citrate buffer, pH 4.5, was injected intraperitoneally. After 10 weeks, the induction was confirmed by a glucose tolerance test using 2 g/kg glucose injected intraperitoneally and subsequent measurement of glycemia by puncturing the rat's tail and retrieving enough blood to be examined with glucose strip in a MediSense Optium capillary glucose meter (Abbott Diabetes Care Inc., Alameda - CA) at 0, 30, 60, 90 and 120 minutes. Animals with hyperglycemia (≥200 mg/dl) after glucose injected intraperitoneally and persistent hyperglycemia after 120 m were considered diabetic. In the other eight, intraperitoneal injection of citrate buffer only, pH 4.5, were administered, and the same glucose tolerance test was performed 10 weeks later.

### Experimental protocol

After glucose tolerance test, which was described above, the diabetic animals were randomly divided into two groups. In one, eight underwent metabolic surgery, comprising the Operated Diabetes Group (DM+OP), and the remaining eight diabetic animals (DM) underwent only clinical follow-up. Finally, the eight animals in which only citrate buffer without Streptozotocin was injected comprised the Control Group (CO). After 12 weeks of life, the rats of the DM+OP group were submitted to an end-to-side duodenojejunostomy to control their glycemic levels.

### Surgical technique

**FIGURE 1:**
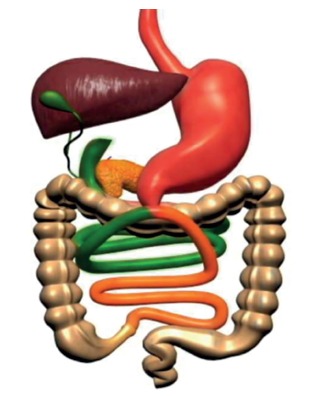
End-to-side duodenojejunostomy with half-and-half biliopancreatic limb for the treatment of type 2 diabetes, described by Marchesini^7^

The experiments were performed under supervision of an experimental animal expert. The anesthetic induction was done with a mixture of oxygen and 0.5% isoflurane through an appropriate facial mask. One only intramuscular dose of 5 mg/kg enrofloxacin (Baytril, Bayer, Shawnee Mission, KS) was administered before the procedure. The animals were submitted to minimal medial laparotomy, duodenum identification and its section 1 cm from the pylorus. The duodenal stump was closed with Prolene 6-0 (Ethicon(r)). The anastomosis point was identified halfway between the duodenojejunal angle and the ileocecal valve. Then, a longitudinal incision was made in the loop, and the end-to-side duodenojejunal continuous anastomosis was performed in a single plane under magnification. This procedure was performed as a modified technique originally described by Rubino^15^ (Figure 1).

### Post-experimental follow-up

The animals were followed for 90 days after the surgery, and their body weight was measured with an electronic digital scale on the day of surgery (day zero) and every 30 days thereafter. On postoperative day 90, the animals of both groups underwent euthanasia by deep anesthesia. Blood, lung, and liver samples were collected.

### Biochemical tests

Venous blood samples were collected through cardiac puncture and centrifuged at 4,000 rpm for 15 m. After centrifugation, the serum was separated and frozen at -80ºC. Serum samples were used to perform the following tests.

#### Lipoperoxidation evaluation

The indirect measurement of lipid peroxidation was performed by measuring TBARS. The concentration obtained was expressed in nmol/mg of protein^3^.

#### Superoxide dismutase activity (SOD)

The activity of this enzyme is defined as its capacity to inhibit a detection system that reacts to O^-2^. The technique of SOD is based on the inhibition of this reaction^9^.

#### Catalase activity

The enzyme catalase catalyzes the decomposition of hydrogen peroxide into water and oxygen. Catalase activity was measured by spectrophotometer, and the concentration was expressed in pmol/g of tissue^1^.

### Statistical analysis

Data are expressed as the mean±standard error. Statistical analyses were performed using the Statistical Package for the Social Sciences software, version 17 (SPSS Inc., Chicago, IL, USA). Differences between the means were assessed using ANOVA followed by Tukey's post hoc or Student Newman-Keuls. The significant level was considered p<0.05.

## RESULTS

### Body weight

**FIGURE 2:**
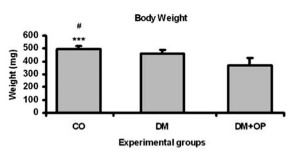
Body weight (g) of the different experimental groups at the end of the experiment with significant difference in the body weight of CO animals compared with animals in the DM ^(#^p<0.01) and DM+OP animals (***p<0.001).

After evaluating the animals' body weight, was observed a significant reduction in the weight of animals from the DM (462±28; p<0.01) and DM+OP (370±55; p<0.001) groups in comparison with the CO (497±25) group animals (Figure 2).

### Oxidative stress - lipid peroxidation

**FIGURE 3:**
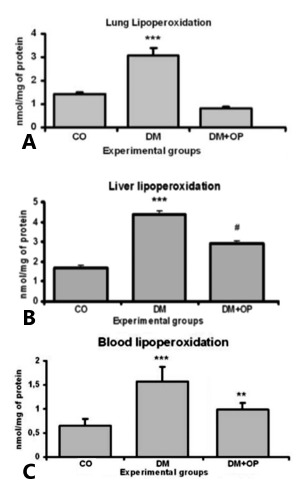
A) Pulmonary lipid peroxidation analysis of TBARS (nmol/mg of protein) with significant increase in DM animals (***p<0.001) compared with CO and DM+OP; B) hepatic lipid peroxidation analysis of TBARS (nmol/mg of protein): a significant increase was observed in the DM animals (***p<0.001) compared with the CO and DM+OP, and a significant difference was observed between the DM+OP and CO animals ^(#^p<0,01); C) blood lipid peroxidation analysis of TBARS (nmol/mg of protein): a significant difference between DM animals (***p<0.001) and CO and DM+OP was observed and a significant difference was observed between DM+OP (**p<0.01) and CO

Using TBARS as a measurement of lipid peroxidation in the lung tissue, was observed a significant increase on it in DM animals (3.072±0.31) compared with CO (1.416±0.07) and DM+OP (0.815±0.08) animals. After duodenojejunostomy, a significant reduction was observed in the lipid peroxidation of pulmonary tissue, demonstrating the protective effect of surgery against oxidative stress (Figure 3A).

In the analysis of hepatic tissue lipid peroxidation, was observed results similar to those in pulmonary tissue; a significant increase in the DM group (4.379±0.17) was observed compared with CO animals (1.699±0.12), and a significant reduction was observed after duodenojejunostomy in the DM+OP animals (2.915±0.13). The DM+OP animals exhibited a significant difference in comparison with the CO (Figure 3B).

In blood tissue, an increase in lipid peroxidation was observed in the DM animals (1.573±0.30) compared with the CO (0.646±0.14), and a significant decrease was observed after surgery was performed in the DM+OP animals (0.993±0.13), further supporting the protective role of surgery in diabetic animals. The DM+OP exhibited a significant difference compared with the CO (Figure 3C).

**FIGURE 4:**
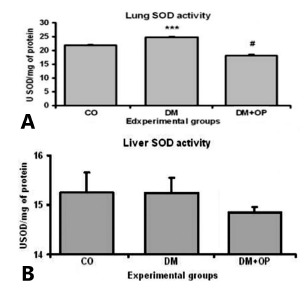
A) Analysis of SOD antioxidant enzymatic activity (USOD/mg of protein) in the pulmonary tissue: a significant difference was observed between the DM (***p<0.001) animals and the CO and DM+OP, and a significant difference was observed between the DM+OP ^(#^p<0.001) and CO; B) analysis of the SOD antioxidant enzymatic activity (USOD/mg of protein) in the hepatic tissue and no significant difference was observed among the groups

 Was evaluated the activity of the antioxidant enzyme SOD in the pulmonary tissue and observed a significant increase in the DM animals (24.792±0.25) compared with the CO (21.776±0.38) and a significant reduction after surgery in the DM+OP (18.077±0.40). These changes demonstrate, in the DM group, the presence of oxidative damage and the subsequent attempt to minimize damage by increasing antioxidant enzyme activity. The same understanding can be applied to the DM+OP animals, in which decreased oxidative damage and decreased SOD enzymatic activity was observed (Figure 4A).

In the hepatic tissue, no significant differences were observed among the DM (15.23±0.31), CO (15.246±0.40) and DM+OP (14.848±0.11) animals. The absence of any difference in hepatic tissue may be due to the superoxide radical anion, which had already been dismuted by the enzyme and converted in hydrogen peroxide (Figure 4B).

**FIGURE 5:**
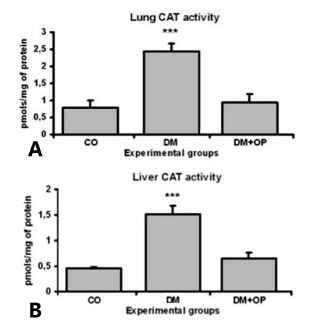
A) Analysis of the antioxidant enzymatic activity of catalase (pmols/mg of protein) in the pulmonary tissue: a significant difference was observed between the DM (***p<0.001) and the CO and DM+OP groups; B) analysis of catalase antioxidant enzymatic activity (pmols/mg of protein) in the hepatic tissue: a significant difference was observed between the DM group (***p<0.001) and the CO and DM+OP groups

 When was analyzed the activity of the antioxidant enzyme catalase in the pulmonary tissue, a significant increase was observed between the DM animals (2.436±0.24) and the CO (0.787±0,21) and DM+OP (0.939±0.24) (Figure 5A). 

This increase confirms an attempt of antioxidant defenses counter stress generated by the important formation of free radicals in diabetes. The animals in the diabetic group in whom duodenojejunostomy was performed exhibited decreased antioxidant enzymatic activity because the animals were already protected against the formation of free radicals, as confirmed through the analysis of lipid peroxidation.

Similar enzymatic activity was observed in hepatic tissue, with a significant increase in the catalase activity in the DM group (1.516±0.17) compared with that in the CO (0.46±0.03) and DM+OP (0.658±0.10) groups (Figure 5B). 

## DISCUSSION

Other authors have previously described finding fact that diabetic animals exhibit decreased weight gain than controls^20^, as this study demonstrated. This decreased weight gain is due to the toxic action of hyperglycemia in the animals during the pubertal period (7-8 weeks), leading to the diminution in the growth rate in this stage. In addition, surgery leads to early weight loss, due either to surgical trauma or to the reduction of early food ingestion, but the weight stabilizes two weeks after surgery. Weight maintenance after surgery in the animals of the DM+OP group verifies the effectiveness of this technique in preventing the progression of obesity, which is typical in obese diabetic patients. Despite the observation of significant weight loss in operated rats in relation to control animals, the animals who were not submitted to surgery remained eutrophic. These data confirm the hypothesis that this surgical technique does not result in significant weight loss, which enables its use in non-obese patients. 

There is growing evidence both in experimental and clinical studies suggesting an important role for oxidative stress in the pathogenesis of DM^8^ ^,^ ^19^ ^,^ ^27^. Studies demonstrated an important increase in TBARS activity in blood and in pulmonary and hepatic tissues in diabetic rats compared with its activity in controls. In addition, duodenojejunostomy resulted in a decrease in TBARS activity to significantly lower levels compared with non-operated diabetic rats. Its measurements in the lung were significantly lower after surgery, even when compared with measurements in non-diabetic controls. These findings demonstrated that surgery reduces lipid peroxidation and decreases the previously high oxidative stress observed in diabetic animals. The liver plays an important role in blood glucose homeostasis. Streptozotocin-induced diabetic rats exhibit an increase in the concentration of lipid peroxidation products such as TBARS, indirect evidence of the intensified free radical production^8^. Reinforcing this model, after duodenojejunostomy, its levels in diabetic rats returned to levels similar to those observed in non-diabetic rats, suggesting an important decrease in lipid peroxidation and the resultant diminution of free radicals. The increase in lipid peroxidation products in the liver indicates that, in DM, the liver is susceptible to lipid peroxidation. Lipid peroxidation in DM is due to the increase in oxidative stress in the cells resulting from the decrease of antioxidant systems. Studies have already demonstrated significant increase in lipid peroxidation in rats exposed to Streptozotocin and have suggested that protective substances, such as gallic acid, may lessen this exaggerated peroxidation. These results suggest the existence of a protective role for antioxidants due to their ability to clear free radicals^12^. A national study with a similar T2DM model did not observed an increase in TBARS in diabetic rats^19^. The authors suggested that the model was insufficient in increasing glycemia to alter TBARS. Nevertheless, the follow-up time (four months) may also have been too short to observe the expected result. In this model, even though fasting glicemia was not sufficiently elevated, after a six month follow-up, an increase in TBARS was evident and highly significant. This may be related to a longer exposure time, as here follow-up was two months longer, which represents a 50% increase above the aforementioned study.

The SOD and catalase activity data obtained in this study are somewhat conflictive. Significantly elevated SOD activity was observed in the lung tissue of diabetic rats compared with controls; however, in hepatic tissue, the values were essentially the same, and operated rats exhibited lower levels of pulmonary SOD than did non-operated rats. However, these results were not exclusive to this study; the literature concerning these studies is conflicting^10^ ^,^ ^16^ ^,^ ^19^ ^,^ ^23^. The SOD enzyme is responsible for the neutralization of superoxide anions, and the reports in the literature of its relationship with DM are conflicting. Although the authors of the abovementioned study on TBARS reported no alteration in SOD activity of rats with Streptozotocin-induced diabetes^19^, which is consistent with other studies^10^ ^,^ ^23^, there is evidence that animals with alloxan-induced diabetes exhibit decreased levels of SOD^23^. In humans, both in type 1 and type 2 DM patients, increases in plasma SOD are observed^18^. In an experimental model of Streptozotocin-induced type 1 diabetes, an increase in pulmonary oxidative stress as well as a reduction in SOD activity was observed in diabetic rats compared with controls^5^. These data confirm the findings of other authors, which demonstrated an increase in oxidative stress and a decrease in SOD activity in the lungs of diabetic rats. These authors also demonstrated an increase in the expression of nitric oxide synthase in the pulmonary tissue of diabetic animals^6^. The other antioxidant enzyme tested in this study, catalase activity, is significantly increased in diabetic animals but is decreased after surgery compared to non-operated diabetic rats. These results were observed both in the liver and in the lung. As previously mentioned, diabetes is a pathologic processes known to be related to an imbalance in Reactive Oxigen Species (ROS) production, such as the hydroxyl radical (HO^-)^, superoxide radical (O_2_^-)^ and H_2_O_2_. Therefore, the cells must be protected from this oxidative lesion by antioxidant enzymes. This is the most likely reason for higher catalase activity observed in diabetic rats compared to controls. Similarly, when oxidative stress decreases after duodenojejunostomy, as observed in this study, the antioxidant activity tends to diminish, constituent with a down-regulation of antioxidant enzymes. This effect has been observed previously in another experimental model, in which a similar increase in catalase activity and in ROS levels were observed in diabetic rats treated with insulin^27^. These data suggest an alteration in oxidant-antioxidant balance in diabetic rats that may be at least partially reestablished through metabolic surgery.

More data from ongoing studies may correlate these findings with intestinal hormone alterations (incretins) and inflammation status.

## CONCLUSION

Duodenojejunostomy was effective in modulating the oxidative stress present in this diabetic rat model. 

## References

[1] Aebi H (1984). Catalase in vitro. Methods Enzymol.

[2] Borges Mde C, Terra GA, Takeuti TD, Ribeiro BM, Silva AA, Terra-Júnior JA, Rodrigues-Júnior V, Crema E. (2015). Immunological evaluation of patients with type 2 diabetes mellitus
submitted to metabolic surgery. Arq Bras Cir Dig.

[3] Buege JA, Aust SD (1978). Microsomal lipid peroxidation. Meth Enzimol.

[4] DATASUS: Ministério da Saúde (2009). Taxa de prevalência de Diabetes Mellitus no Brasil.

[5] Forgiarini LA, Kretzmann NA, Porawski M, Dias AS, Marroni MA (2009). Experimental diabetes mellitus oxidative stress and changes in lung
structure. J Bras Pneumol.

[6] Hürdag C, Uyaner I, Gürel E, Utkusavas A, Atukeren P, Demirci C (2008). The effect of alpha-lipoic acid on NOS dispersion in the lung of
streptozotocin-induced diabetic rats. J Diabetes Complications.

[7] Marchesini JC (2007). End-to-side duodeno-jejunostomy with half-and-half biliopancreatic
limb for the treatment of type 2 diabetes a proposal for a simpler
technique. Obes Surg.

[8] Maritim AC, Sanders RA, Watkins JB (2003). Diabetes, oxidative stress, and antioxidants a review. J Biochem Mol Toxicology.

[9] Misra HP, Fridovich I (1972). The role of superoxide anion in the autoxidation of epinephrine and a
simple assay for superoxide dismutase. J Biol Chem.

[10] Muruganandan S, Gupta S, Kataria M, Lal J, Gupta PK (2002). Mangiferin protects the streptozotocin-induced oxidative damage to
cardiac and renal tissues in rats. Toxicology.

[11] Oliveira LF, Tisott CG, Silvano DM, Campos CM, Nascimento RR (2015). Glycemic behavior in 48 hours postoperative period of patients with
type 2 diabetes mellitus and non diabetic submitted to bariatric
surgery. Arq Bras Cir Dig.

[12] Punithavathi VR, Stanely Mainzen Prince P, Kumar MR, Selvakumari CJ (2011). Protective Effects of Gallic Acid on Hepatic Lipid Peroxide
Metabolism, Glycoprotein Components and Lipids in Streptozotocin-Induced Type II
Diabetic Wistar Rats. J Biochem Molecular Toxicology.

[13] Rubino F, Gagner M (2002). Potential of surgery for curing type 2 diabetes
mellitus. Ann Surg.

[14] Rubino F, Kaplan LM, Schauer PR, Cummings DE (2010). The Diabetes Surgery Summit consensus conference recommendations for
the evaluation and use of gastrointestinal surgery to treat type 2 diabetes
mellitus. Ann Surg.

[15] Rubino F, Marescaux J (2004). Effect of duodenal-jejunal exclusion in a non-obese animal model of
type 2 diabetes a new perspective for an old disease. Ann Surg.

[16] Sailaja Devi MM, Suresh Y, Das UN (2000). Preservation of the antioxidant status in chemically-induced Diabetes
mellitus by melatonin. J Pineal Res.

[17] Sampaio-Neto J, Nassif LS, Branco-Filho AJ, Bolfarini LA, Loro LS, de Souza MP, Bianco T (2015). External validation of the diarem score as remission predictor of
diabetes mellitus type 2 in obese patients undergoing roux-en-y gastric
bypass. Arq Bras Cir Dig.

[18] Seghrouchni I, Drai J, Bannier E (2002). Oxidative stress parameters in type I, type II and insulin-treated
type 2 Diabetes mellitus insulin treatment efficiency. Clin Chim Acta.

[19] Sinzato YK, Lima PH, Campos KE, Kiss ACI, Rudge MV, Damasceno DC (2009). Neonatally-induced diabetes lipid profile outcomes and oxidative
stress status in adult rats. Rev Assoc Med Bras.

[20] Takada J, Machado MA, Peres SB (2007). Neonatal streptozotocin-induced diabetes mellitus a model of insulin
resistance associated with loss of adipose mass. Metabolism.

[21] Takeuti TD, Terra GA, da Silva AA, Terra JA, da Silva LM, Crema E (2014). Effect of the ingestion of the palm oil and glutamine in serum levels
of GLP-1, PYY and glycemia in diabetes mellitus type 2 patients submitted to
metabolic surgery. Arq Bras Cir Dig.

[22] Thaler JP, Cummings DE (2009). Hormonal and Metabolic Mechanisms of Diabetes Remission After
Gastrointestinal Surgery. Endocrinology.

[23] Tormo MA, Romero de Tejada A, Morales I (2004). Orally administered tryptophan and experimental type 2
diabetes. Mol Cell Biochem.

[24] Unwin N, Gan D (2010). Whiting D The IDF Diabetes Atlas: providing evidence, raising
awareness and promoting action. Diabetes Res Clin Pract.

[25] Wietzycoski CR, Von Diemen V, Trindade MRM, Rohde L, Osvaldt AB (2010). Cirurgia Metabólica. Condutas em Cirurgia Digestiva.

[26] Wild S, Roglic G, Green A, Sicree R, King H (2004). Global prevalence of diabetes estimates for the year 2000 and
projections for 2030. Diabetes Care.

[27] Wohaieb SA, Godin DV (1987). Alterations in free radical tissue defense mechanisms in
streptozotocin-induced diabetes in rat Effects of insulin
treatment. Diabetes.

[28] World Health Organization (1998). The World Health Report.

